# Reliability of high-resolution ultrasound and magnetic resonance arthrography of the shoulder in patients with sports-related shoulder injuries

**DOI:** 10.1371/journal.pone.0222783

**Published:** 2019-09-23

**Authors:** Georg J. Wengert, Marc Schmutzer, Hubert Bickel, Mircea-Constantin Sora, Stephan H. Polanec, Micheal Weber, Claudia Schueller-Weidekamm

**Affiliations:** 1 Department of Biomedical Imaging and Image-guided Therapy, Medical University of Vienna, Vienna, Austria; 2 Morphology Centre, Medical Faculty, Sigmund Freund University, Vienna, Austria; Brigham and Women's Faulkner Hospital, UNITED STATES

## Abstract

**Introduction:**

The shoulder, a very complex joint, offers a wide range of pathologies. Intraarticular abnormalities and rotator cuff injuries are mainly assessed and diagnosed by magnetic resonance arthrography (MRA). In contrast to this well-established gold standard, high-resolution ultrasound (US) offers an additional easy and excellent modality to assess the shoulder joint. Therefore, the purpose of this study was to evaluate in which anatomic structures and pathologies comparable results of US and MRA could be achieved.

**Materials and methods:**

In this IRB-approved prospective study 67 patients with clinically suspected labral lesions, rotator cuff rupture, or injury of the long head of the biceps (LHB) tendon were enrolled. Each participant was examined with high resolution US, and directly followed by MRA at 3 Tesla with a standard sequence protocol. To evaluate the agreement of the diagnostic performance between US and MRA a weighted kappa statistic was used.

**Results:**

Both of the investigated modalities yielded a moderate to almost perfect agreement in assessing a wide range of shoulder joint pathologies. For the rotator cuff, consistency was found in 71.64% for the supraspinatus tendon, in 95.52% for the infraspinatus tendon, in 83.58% for the subscapularis tendon, and in 98.51% for the teres minor tendon.

The diagnostic accuracy between both modalities was 80.60% for the LHB tendon, 77.61% for the posterior labroligamentous complex, 83.58% for the acromioclavicular joint, and 91.04% for the assessment of osseous irregularities and impaction fractures.

**Conclusions:**

High resolution US is a reliable imaging modality for the rotator cuff, the LHB tendon, and the acromioclavicular joint, so for these structures we recommend a preference for US over MRA based on its diagnostic accuracy, comfortability, cost effectiveness, and availability. If the diagnosis remains elusive, for all other intraarticular structures we recommend MRA for further diagnostic assessment.

## Introduction

The glenohumeral joint is a complex articulation that provides the greatest range of motion of any joint in the human body [1]. The drawback of this great mobility is an increased risk of instability, repetitive injury, and chronic pathology [2, 3]. Therefore, tailored diagnostic imaging in combination with clinical examination is essential for management and treatment planning [4]. Conventional radiographs serve as the baseline imaging modality for shoulder disorders. Magnetic resonance imaging (MRI) and high-resolution ultrasound (US) are promising modalities for future assessment of various shoulder joint disorders and pain syndromes [5–7]. Intraarticular application of contrast agent, as applied in magnetic resonance arthrography (MRA), provides an additional benefit by better visualization of intraarticular structures compared to conventional MRI of the shoulder [7, 8]. The use of US in the shoulder is a widely available and cost-effective alternative to expensive and complex MRI and MRA examinations with long waiting times, and is usually recommended in first instance together with radiography [9].

Selection of the appropriate diagnostic method to assess shoulder joint instabilities, extrinsic and intrinsic impingement requires a systematic clinical examination of the shoulder. US is excellent suitable for the dynamic evaluation of subacromial disorders, pathologies of the rotator cuff, and injuries on the long head of the biceps (LHB) tendon. MRA is suitable to assess the labroligamentous complex, the cartilage, and the bone marrow in addition to major anatomic structures.

In this study we evaluated which structures and pathologies of the shoulder showed comparable results when examined with high-resolution US and MRA in patients with sports related injuries.

## Materials and methods

### Study design

Participants for this IRB approved prospective study were recruited from October 2011 to April 2014. In total, 67 patients with clinically suspected lesions, rotator cuff rupture, or injury of the LHB and biceps pulley were included. After written informed consent, MRI examinations were performed within one hour after a previous diagnostic high-resolution ultrasound US examination and after US guided intra-articular contrast media injection. Exclusion criteria were contraindications for MR imaging, claustrophobia, and age under 18 years.

### High-resolution ultrasound

The US examination was based on the standardized guidelines of the European Society of Musculoskeletal Radiology (ESSR) [10, 11]. Each patient was examined in real time with the use of a GE Logiq E9 scanner (General Electric Healthcare Company, GB) and a variable high-frequency linear-array transducer with 12 to 15 Megahertz (MHz) and a high frequency hockey-stick transducer (18 MHz).

A radiologist with 15 years of experience in musculoskeletal US performed the non-invasive US examination while facing the seated patient.

First, the LHB tendon was investigated in longitudinal and transverse plane. In the transverse plane the LHB was examined from the intra-capsular origin to its course within the bicipital groove down to the muscular transition with the arm slightly externally rotated close to the chest and the elbow flexed in the right angle. In external rotation, a possible medial subluxation of the long head of the biceps tendon can be detected and is suggestive of laxity or tear of the transverse ligament that is formed by the superficial fibres of the subscapularis tendon. Full thickness tears of the superior portion of the subscapularis tendon allows inferior subluxation of the LHB tendon. Another possibility for LHB tendon subluxation is a tear of the biceps pulley, which consists of the superior glenohumeral ligament and the coracohumeral ligament.

Further external rotation of the arm allows the assessment of the subscapularis tendon and muscle. Examination of the supraspinatus tendon was performed in a modified crass position with the hand placed on the lateral iliac crest and with the elbow flexed to extend the supraspinatus tendon, this allowed a simplified biceps pulley visualization with little patient discomfort [12]. Imaging of the infraspinatus and teres minor tendons were made in the previous position with the arm slightly internally rotated. The surface of the humeral head was investigated for osseous irregularities at the tendon insertion and for impaction fracture at the posterosuperior aspect of the humeral head.

The passive abduction of the arm demonstrated the slipping of the supraspinatus tendon below the acromion to demonstrate subacromial impingement. To examine the infraspinatus and teres minor muscles, and the postero-inferior labral complex the transducer was moved in the longitudinal and the transversal planes on the dorsal aspect of the shoulder. Paralabral cysts or joint effusion undermining the labrum in particular in internal and external rotation was highly suggestive of labral tear. The remaining labroligamentous complex could not be assessed on US due to limited visibility of these structures. The acromioclavicular joint was examined from the superior aspect by shifting the transducer slightly anteriorly and posteriorly in the coronal plane.

Diagnostic US examination of the shoulder was followed by intraarticular contrast media injection for subsequent MRA examination. The patient’s position was changed from erect to prone. The arm was placed in an outstretched position with internal rotation next to the body to expand the posterior part of the glenohumeral joint. Approximately 1:200 diluted 15–20 ml gadolinium-based contrast media (Artirem©, Guerbet) was injected from a posterior approach under US guidance into the glenohumeral joint. The tip of the 21 G needle and the dilatation of the joint capsule were checked under US control to avoid extravasation.

### Magnetic resonance imaging

MR Arthrography (MRA) was performed using a 3.0 T unit (Siemens Tim Trio; Erlangen, Germany) with an actively shielded gradient system with a maximum gradient strength of 45 mT/m and a dedicated 4-channel shoulder coil (In Vivo, Pewaukee, WI). Each patient was examined in a supine position with the arm in slight external rotation. The images were acquired using the following standard imaging protocol including a fat-suppressed proton density weighted sequence in the oblique coronal plane oriented along the scapula (TR/TE 3500/34ms, 23 slices, voxel size 0.7x0.6x3.0mm, TA 3:49min), T1-weighted turbo spin echo sequences with and without fat-suppression in the oblique coronal plane (TR/TE 734/21ms, 23 slices, voxel size 0.8x0.6x3.0mm, TA 3:58min), a fat-suppressed T1-weighted turbo spin echo sequence in the transversal plane (TR/TE 767/11ms, 25 slices, voxel size 0.7x0.5x3.0mm, TA 3:47min), and a fat-saturated T1-weighted turbo spin echo sequence in the oblique sagittal plane oriented along the glenoid (TR/TE 853/11ms, 30 slices, voxel size 0.7x0.5x3.0mm, TA 5:29min). An additional fat-saturated T1-weighted sequence in abduction and external rotation (ABER) position using a dedicated 4-channel body array coil (Siemens AG, Munich, GER) was acquired (TR/TE 957/23ms, 30 slices, voxel size 0.5x0.4x3.0mm, TA 7:14min). This additional sequence can provide a better visualization and assessment of the anterior inferior labro-ligamentous complex and of the supraspinatus and the biceps anchor [13]. The total time for positioning and scanning in ABER position lasted no longer than five minutes.

### Imaging analysis

Images were evaluated by one radiologist with 15 years of experience in musculoskeletal imaging on a picture archiving and communicating workstation (PACS, IMPAX EE, Agfa HealthCare GmbH, Germany). For all US and MRA examinations the supraspinatus tendon, the infraspinatus tendon, the subscapularis tendon, the teres minor tendon, and the LHB tendon were evaluated with the following classification system: unremarkable (no abnormalities), partial rupture, complete rupture, and degeneration. The biceps pulley, the superoanterior, anteroinferior and posteroinferior labroligamentous complex, as well as the superior, medial, and inferior glenohumeral ligaments had been additionally considered in the MRA reports and were classified, respectively [13–15]. In addition, degenerative changes of the acromioclavicular joint, bone marrow edema, osseous irregularities such as impaction fractures of the humeral head (Hill-Sachs) and Bankart Lesions were assessed on MRA.

### Statistical analysis

Statistical analysis was performed using IBM SPSS Statistics for Windows version 22, Armonk, NY. Patient age was described using mean +/- SD. For previous trauma, dislocation of the shoulder, and recreational sports, absolute numbers and percentages were used to summarize the study sample. To abstract the agreement of the diagnostic performance between MRA and US we used a weighted kappa statistic with the interpretation of kappa according to Viera et al. [16].

## Results

In total 67 patients, 47 males and 20 females, with a mean age of 39.6 years (range 18 to 71 years) fulfilled the inclusion criteria of this study. Previous non-classified trauma or repetitive shoulder dislocation was reported in twelve patients, and in an additional 28 patients a single event of shoulder dislocation. Specified recreational sports identified included swimming, volleyball, tennis, golf, shooting, running, boxing, and snowboarding however, in the vast majority, no specific sport activity was declared.

Table 1 gives a summary of the investigated anatomical structures and pathologies found on MRA and US.

**Table 1:** Descriptive results of the assessed anatomical structures and categorization of no abnormal or pathologic findings on MRA and US of the shoulder.

		MRA	US
Structure	Category		
Supraspinatus Tendon	Unremarkable	13	19
	Partial Rupture	27	23
	Complete Rupture	11	7
	Degeneration	16	18
Infraspinatus Tendon	Unremarkable	63	64
	Partial Rupture	2	2
	Complete Rupture	0	0
	Degeneration	2	1
Subscapularis Tendon	Unremarkable	50	55
	Partial Rupture	7	4
	Complete Rupture	2	2
	Degeneration	8	6
Teres Minor Tendon	Unremarkable	66	67
	Partial Rupture	0	0
	Complete Rupture	0	0
	Degeneration	1	0
Long Head of the Biceps Tendon	Unremarkable	47	60
	Partial Rupture	2	0
	Complete Rupture	0	0
	Degeneration	18	7
Posterior Labroligamentous Complex	Unremarkable	54	52
	Partial Rupture	1	0
	Complete Rupture	6	1
	Degeneration	6	14
Acromioclavicular Joint	Unremarkable	42	49
	Degeneration	25	18
Osseous Changes	Unremarkable	19	23
	Present	48	44
	Not Present	0	0

### Supraspinatus tendon

**Fig 1:**
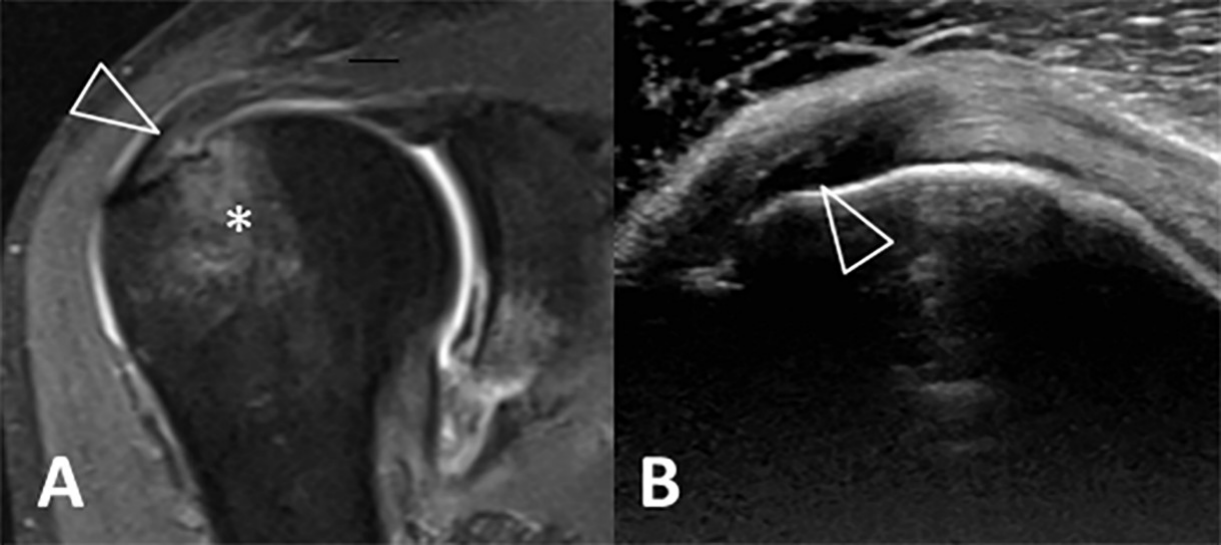
Coronal fat saturated proton density MRA (A) and Ultrasound (B) of the right shoulder of a 53-year-old male patient. There is an articular-sided hyperintense signal change at the supraspinatus tendon insertion (A, open arrow); on the corresponding ultrasound image of the same patient, there is an area of low echogenicity at the same location of the tendon (B, open arrow). In addition, extensive bone marrow edema in the humeral head is seen on MRA (A, asterisk). Additional finding of thickening and narrowing of the axillary recess on the MRA raises the possibility of adhesive capsulitis.

The diagnostic agreement between high-resolution US and MRA of the shoulder for the supraspinatus tendon was substantial (k = 0.609) (Fig 1). In both modalities the same pathology was assessed in 71.64%.

Of eight patients with an unremarkable US examination, six partial ruptures of the supraspinatus tendon, and two tendinopathies were seen on MRA. In four out of five patients with partial thickness tear on US, a complete rupture was seen on MRA. On the other hand, the partial-thickness tear of one patient on MRA was misinterpreted as full-thickness tear with US. Tendinosis seen on US was interpreted as unremarkable in five patients, as a partial-thickness tear in two patients, and as a full thickness tear in one patient on MRA.

### Infraspinatus tendon

The agreement between MRA and US for the infraspinatus tendon was moderate (k = 0.554), but with a diagnostic accuracy of 95.52% of the assessed findings.

In both modalities a partial thickness tear was found in two patients, whereas US missed one tendinosis of the infraspinatus tendon.

### Subscapularis tendon

Moderate agreement between MRA and US was calculated for pathologies of the subscapularis tendon, (k = 0.556), with a diagnostic accuracy of 83.58%.

No abnormalities were found on 55 out of 67 cases assessed with US. On MRA, 50 patients were unremarkable. In five patients, tendinosis was seen on MRA that appeared to be unremarkable on US. On the other hand, in three patients, tendinosis was seen on US and not on MRA.

### Teres minor tendon

The diagnostic agreement between MRA and US for the teres minor tendon was perfect. The calculated diagnostic accuracy was 98.51% as tendinosis was missed in one patient on US.

### Long head of the biceps tendon

Moderate agreement (k = 0.436) was present for the long head of the biceps tendon with a diagnostic accuracy of 80.60% between MRA and US.

With MRA it was possible to identify additional tendinosis in eleven patients and two partial-thickness tears.

### Posteroinferior labroligamentous complex

A fair to moderate agreement was calculated for pathologies of the posteroinferior labro-ligamentous complex (k = 0.407); however, the diagnostic accuracy was 77.61% between MRA and US.

Ultrasound detected additional degenerative changes of the posteroinferior labroligamentous complex in eight patients compared to MRA. In view of MRA is considered as gold standard, these findings have to be interpreted as false positive.

US was superior to MRA in detecting degenerative changes of the posteroinferior labroligamentous complex in an additional eight patients, whereas this anatomic structure was unremarkable on MRA in these patients.

### Acromioclavicular joint

**Fig 2:**
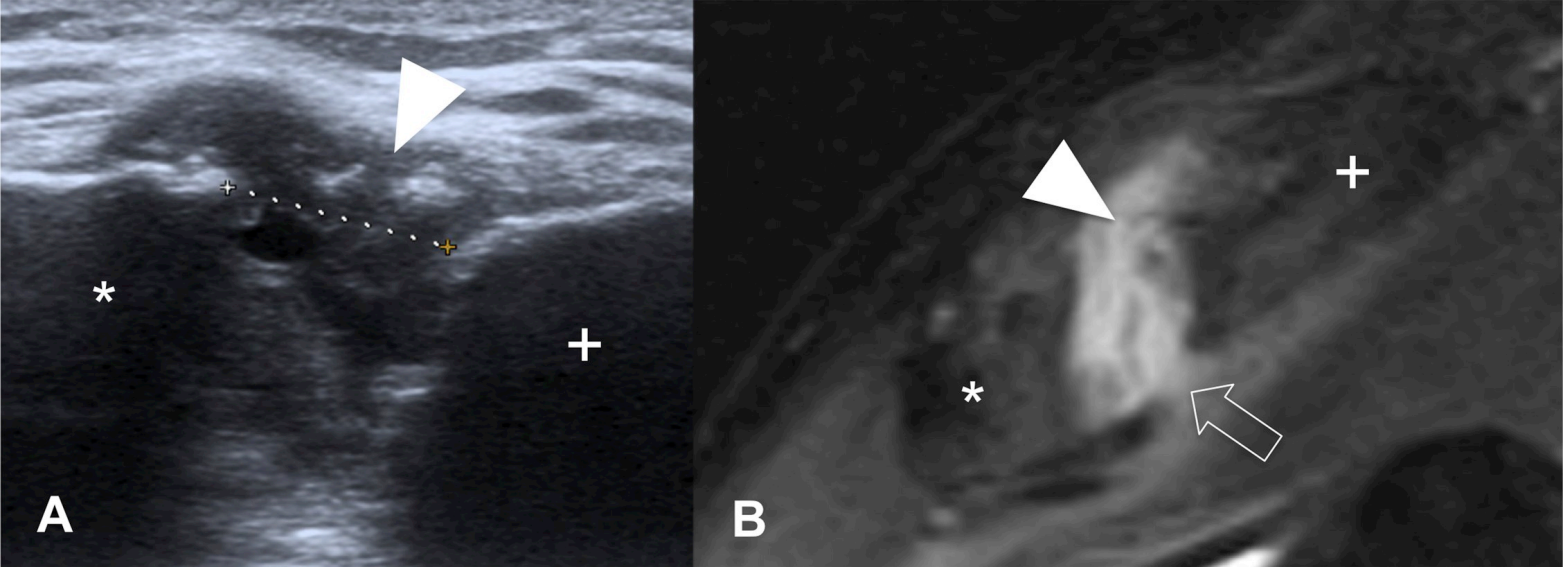
Ultrasound (A) and coronal fat saturated proton density MRI (B) of the right acromioclavicular joint in a 36-year-old male after a bicycle accident. In both modalities, the superior ACL (arrowhead) is completely absent. There is also a rupture of the inferior ACL (open arrow), which can only be seen on MRI. Joint effusion is present; acromion (*); clavicle (+).

The diagnostic accuracy of the acromioclavicular joint pathologies between MRA and US was 83.58%, with a substantial agreement between both modalities (k = 0.636) (Fig 2).

Additional degenerative changes of the acromioclavicular joint were identified on fat-suppressed proton density weighted MR sequences in seven patients that were unremarkable on US.

### Osseous changes

The assessment of osseous irregularities and bone impaction of the shoulder joint resulted in a substantial agreement between MRA and US (k = 0.793). The diagnostic accuracy was 91.04% for the assessed findings.

Compared to US, four additional osseous irregularities at the insertion of the rotator cuff tendons were depicted on MRA and were associated with enthesopathic changes.

Impaction fracture at the posterosuperior aspect of the humeral head, highly suggestive of Hill-Sachs lesion and commonly seen in patients with anterior shoulder dislocation, were found in 16 patients on both, MRA and US. The different additional shoulder pathologies, only seen on MRI, are listed in Table 2.

**Table 2:** Descriptive results of additional shoulder pathologies only seen on MRA.

	Pathology on MRA	
Bankart Lesion		10/67
Reversed Bankart Lesion		1/67
SLAP Lesion		7/67
Bone Marrow Edema		30/67
Biceps pulley		22/67
Superior glenohumeral ligament		13/22
Superior insertion of the subscapularis tendon		5/22
Coracohumeral ligament		1/22
Combined injuries of superior glenohumeral and coracohumeral ligaments		2/22
Combined injuries of superior glenohumeral ligament and superior insertion of the subscapularis tendon		1/22

**Fig 3:**
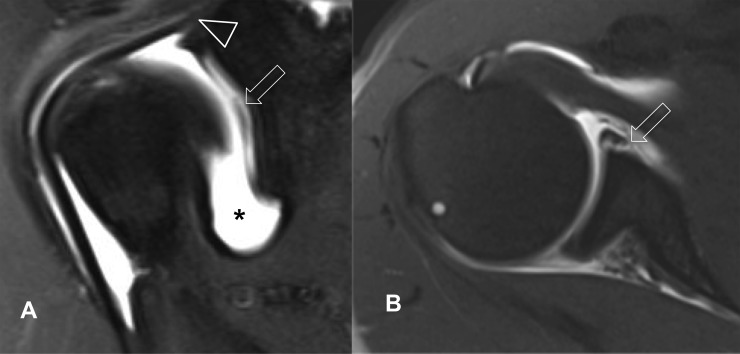
Coronal fat saturated proton density (A) and axial fat saturated T1-weighted (B) MRA of the right shoulder in a 45-year-old male tennis player. There is an extensive superior labral anterior to posterior (SLAP) tear (open arrow), while the biceps anchor (arrowhead) is still intact. Intraarticular contrast agent (*) has been applied prior to the examination.

Ten Bankart lesions, one reversed Bankart lesion, seven SLAP lesions, and 30 patients with bone marrow edema could be identified only with MRA (Fig 3). Pathologies of the biceps pulley that consist of the superior glenohumeral and the coracohumeral ligaments, the insertion of the subscapularis tendon, and the medial and inferior glenohumeral ligaments could only be assessed with MRA. On MRA, biceps pulley injuries were found in 22 out of 67 cases, consisting of 13 full-thickness tears of the superior glenohumeral ligament, 5 injuries of the subscapularis tendon insertion, and a single rupture of the coracohumeral ligament. Combined injuries of the superior glenohumeral and the coracohumeral ligaments were present in two patients, and combined injury of the superior glenohumeral ligament and the distal attachment of the subscapularis tendon was present in one case on MRA.

## Discussion

The investigated diagnostic assessment of shoulder joint disorders in patients with sports related injuries, carried out with high-resolution US and MRA demonstrated moderate to substantial correlation and a diagnostic accuracy of 71.64% - 98.51%. Especially in the assessment of pathologies of the rotator cuff a good agreement was achieved.

Several studies have shown that US of the shoulder is a reliable tool as an initial examination technique for patients with acute or chronic shoulder pain [6, 17–19]. Major benefits of US as an attractive diagnostic method for shoulder joint disorders are the high availability, short investigation time, possibility of bilateral examination, and it is also less expensive compared to MRI. In postoperative patients in whom metal artefacts could reduce image quality or in claustrophobic patients, US should be preferred. Some pathologies, such as, lateral impingement can only be assessed by dynamic US examination. On the other hand, the accuracy of US depends on the experience of the examining sonographer and thus requires a longer learning curve.

MRI, and especially MRA, of the shoulder is a standard examination in the daily clinical routine of the musculoskeletal radiologist, providing high sensitivity and specificity for overall evaluation and detection of shoulder pathologies [8, 20, 21]. However, high costs of MRI/MRA and limited availability, especially in rural areas or provincial hospitals often demand alternative imaging modalities. Opposing the dynamic ultrasound assessment of the LHB tendon, MRA enables the evaluation of the intraarticular portion of the LHB tendon, in particular at the biceps anchor. However, luxation of the LHB tendon can be missed on MRA depending on positioning of the shoulder during the MR examination. Joint effusion is missed on MRA but can easily be assessed on US at the axillary recess, at the posterior glenohumeral recess or along the LHB tendon.

The choice of the imaging modality is strongly based on clinical examination and differs according to suspected internal or external impingement. External osseous impingement is usually assessed by radiography and associated bursitis is seen on dynamic US examination, by possible bulging of the subacromial/subdeltiod bursa during abduction. Labral and cartilage lesions and malpositioning of the humeral head together with capsular and/or ligamentous thickening seen on MRA raise the possibility of internal impingement [2, 22].

The results of our study are in agreement with other published results that showed a reliable diagnosis of specified shoulder pathologies. Recently, Lenza et al. published a systematic review for assessment of the diagnostic accuracy for MRI, MRA, and US in the detection of rotator cuff tears. Unfortunately significant methodological issues limited the validity of the investigations, but they concluded a similar accuracy for the three different diagnostic methods in the detection of full-thickness tears of the rotator cuff, but a poor sensitivity of MRI and US in detection of partial thickness tears [23].

A meta-analysis of de Jesus et al. also compared the detection of partial and full-thickness tears of the rotator cuff with MRI, MRA, and US with similar results to ours. For both pathologies, MRA showed the highest sensitivity and specificity, whereas MRI and US were comparable to each other, but were not as good as MRA [24]. Our results showed an 83.5% diagnostic agreement for rotator cuff pathologies when comparing US to MRA. The partial thickness tears detected in our study were articular sided located. These partial thickness tears were also well depicted on MRA secondary to the intra-articular contrast media that provides a better contrast between the tendon fibers and the contrast media accumulation within the defect. No intra-tendinous partial thickness tears were seen on US of fat-suppressed proton density MR sequences.

Differences in the diagnostic performance for the detection of superior labrum anterior to posterior (SLAP) lesions between MRA and MRI were recently investigated in a systematic review and meta-analysis by Arirachakaran et al. [8]. They found a significant superiority of MRA over MRI in standard positioning and an additional benefit in the ABER position. Furthermore, MRI with higher field strengths (3.0 vs. 1.5 Tesla) was comparable to MRA in the diagnosis of SLAP lesion detection.

A study published by Jonas et al. showed a poor sensitivity (58%) of MRA secondary to a high number of false negative assessments of the anterior labral tears in patients with shoulder dislocation [25]. These findings show that correct MR arthrogram protocols and an awareness of possible missed chronic labral tears is essential. The results also clarify that interpretation of MR images is operator-dependent.

Major limitations of our study are the relatively small number of patients and the small number of pathologies in most of the assessed anatomic structures, with the highest number of pathologies in the rotator cuff. Another limitation is the lack of correlating the findings with arthroscopy, as only a very small fraction underwent surgery. In our study, MRA was considered as the gold standard. Interobserver agreement could not be obtained as only one radiologist evaluated the findings. The greatest strength of our study is the assessment of the anatomic structures with US and MRA on the same day. This excludes an interval re-injury or healing of the injuries between the two different examinations.

In conclusion, high-resolution US of the shoulder can be used as a reliable diagnostic method for assessment of the rotator cuff, the LHB, the posterior labrum, and the AC joint compared to MRA. The fact that US represents a readily available, cost-effective, and patient-friendly modality with the additional benefit of dynamic examination should increase the use of US in conjunction with clinical examination prior to MRA for the stated structures.
